# Longitudinal genome-wide DNA methylation changes in response to kidney failure replacement therapy

**DOI:** 10.1038/s41598-021-04321-5

**Published:** 2022-01-10

**Authors:** Anna Witasp, Karin Luttropp, Abdul Rashid Qureshi, Peter Barany, Olof Heimbürger, Lars Wennberg, Tomas J. Ekström, Paul G. Shiels, Peter Stenvinkel, Louise Nordfors

**Affiliations:** 1 Division of Renal Medicine, Department of Clinical Science, Intervention and Technology, Karolinska Institutet, Karolinska University Hospital, M99, 141 86 Stockholm, Sweden; 2 Center for Molecular Medicine, Karolinska Institutet, Stockholm, Sweden; 3 Department of Molecular Medicine and Surgery, Karolinska Institutet, Stockholm, Sweden; 4 Division of Transplantation Surgery, Department of Clinical Science, Intervention and Technology, Karolinska University Hospital, Stockholm, Sweden; 5 College of Medical, Veterinary and Life Sciences Institute of Cancer Sciences, University of Glasgow, Glasgow, UK

**Keywords:** Molecular biology, Epigenetics, DNA methylation, Medical research, Translational research

## Abstract

Chronic kidney disease (CKD) is an emerging public health priority associated with high mortality rates and demanding treatment regimens, including life-style changes, medications or even dialysis or renal transplantation. Unavoidably, the uremic milieu disturbs homeostatic processes such as DNA methylation and other vital gene regulatory mechanisms. Here, we aimed to investigate how dialysis or kidney transplantation modifies the epigenome-wide methylation signature over 12 months of treatment. We used the Infinium HumanMethylation450 BeadChip on whole blood samples from CKD-patients undergoing either dialysis (n = 11) or kidney transplantation (n = 12) and 24 age- and sex-matched population-based controls. At baseline, comparison between patients and controls identified several significant (P_FDR_ < 0.01) CpG methylation differences in genes with functions relevant to inflammation, cellular ageing and vascular calcification. Following 12 months, the global DNA methylation pattern of patients approached that seen in the control group. Notably, 413 CpG sites remained differentially methylated at follow-up in both treatment groups compared to controls. Together, these data indicate that the uremic milieu drives genome-wide methylation changes that are partially reversed with kidney failure replacement therapy. Differentially methylated CpG sites unaffected by treatment may be of particular interest as they could highlight candidate genes for kidney disease per se.

## Introduction

Chronic kidney disease (CKD) is becoming increasingly common worldwide^1^. As CKD progresses, the patient approaches kidney failure (KF), defined as a glomerular filtration rate (GFR) < 15 ml/min (i.e. CKD stage 5)^2^. When GFR reaches 5–10 ml/min, kidney failure replacement therapy (KFRT) with either peritoneal dialysis, haemodialysis or kidney transplantation (KTx), is required for patient survival. Treatment with KTx is usually preferable as it results in better quality of life and long-term patient survival^3^. Around three million patients are currently receiving KFRT, a number expected to increase to between 5 and 10 million by 2030^4^, but the biological effects of KFRT are far from fully explored.

In addition to the strain of lifelong KFRT, CKD patients commonly suffer from reduced quality of life and various comorbidities, the most prominent being premature cardiovascular disease (CVD), frailty, osteoporosis, protein energy wasting and infectious complications. The CVD prevalence in patients with CKD is disproportionately high compared to age-matched healthy individuals^5,6^, suggesting that CKD triggers early vascular ageing processes^7^. Vascular ageing and CVD are multifaceted conditions with many underlying causes and complex disease patterns. Among the many risk factors for athero- and arteriosclerosis, inflammation features prominently^8^. Inflammation, as part of a senescence associated secretory phenotype (SASP), also appears to be connected to premature cellular ageing, cellular senescence and apoptosis; processes which have been proposed as potential causative mechanisms for increased vascular calcification in arterial tissue^7–9^. Indeed, dialysis treatment per se induces apoptosis in arteries with a subsequent increase in CVD risk^10–12^, as well as SASP factors that correlate with increased vascular stiffness and coronary artery calcification^13^.

Patients that progress to KF are often exposed to a toxic uremic milieu over a long period of time. The impact of this toxic milieu on allostatic load remains largely unexplored, as is its impact on fundamental cellular processes. This includes the dynamic epigenetic regulation of chromatin and gene transcription. DNA methylation, i.e. cytosine methylation in the CpG dinucleotide context, is part of the epigenetic machinery that regulates cell type-specific regulation of gene expression, and provides a mechanism for altering the activity of the genome in response to various environmental stimuli^14^. Often, but not always, DNA methylation results in transcriptionally inactive chromatin. This regulatory function is essential for normal development and differentiation of cells and tissues but is also known to play a role in many pathological processes driving complex disease, such as tumorigenesis^15^, neuropsychiatric disorders^16^, diabetes mellitus (DM)^17^, rheumatoid arthritis^18,19^ and atherosclerosis^20^. Maladaptive DNA methylation in the context of the CKD spectrum has become increasingly recognized^21–27^ and we have previously shown an association between differential DNA methylation and CVD in patients with CKD^28^.

It remains unclear whether alterations in DNA methylation present in the uremic milieu can be reversed by KFRT, and if so, to what extent. To investigate this, we quantitatively identified genome-wide CpG methylation in whole-blood from KF patients either initiating dialysis or undergoing KTx. In a cross-sectional design, patients at baseline (immediately before KFRT initiation) were compared to age-matched population-based control subjects and longitudinal DNA methylation changes were subsequently investigated in the same patients following 12 months of KFRT.

## Results

### Demographics and clinical characteristics at baseline

Patient and control characteristics at baseline can be found in Table 1*.* One patient in the dialysis group was identified as an outlier (regarding signal strength and detectable probe ratio) in the initial lumi analysis and was therefore excluded, resulting in a total of 23 patients (11 dialysis and 12 KTx patients) in the study. Compared to the control group, incident dialysis patients had lower serum albumin (34 vs 40 g/l; p = 0.012) and cholesterol (4.3 vs 5.1 mmol/l; p = 0.04) but higher high-sensitivity C-reactive protein (hsCRP) (1.8 vs 0.6 mg/l; p = 0.02) and interleukin-6 (IL-6) (5.0 vs 1.2 pg/ml; p = 0.0002). Patients undergoing KTx had lower serum albumin (36 vs 40 g/l; p = 0.0005) and higher triglycerides (1.8 vs 1.0 mmol/l; p = 0.02) and were slightly younger (48 vs 52 years; p = 0.042) than controls. Some differences were noted between the two patient groups at baseline. Incident dialysis patients had significantly higher serum IL-6 (5.0 vs 1.0 pg/ml; p = 0.0003) and high-density lipoprotein (HDL) (1.5 vs 1.3 mmol/l; p = 0.04), as well as lower leukocyte (7.5 vs 8.8 10^9^/l; p = 0.03) and neutrophil count (4.1 vs 6.7 10^9^/l; p = 0.03) than patients undergoing KTx.

**Table 1:** Clinical and demographic characteristics of KF patients and controls.

	Controls	Kidney transplantation group	Kidney transplantation group	Incident dialysis group	Incident dialysis group	p-value
	Baseline	Baseline	1 year	Baseline	1 year	
	N = 24	N = 12	N = 12	N = 11	N = 11	
**Clinical characteristics**						
Age (years)	51.5 (46.5–56.0)	48.0 (44.5–50.0)	49.0 (45.5–51.0)	48.0 (43.0–54.0)	49.0 (44.0–55.0)	0.27
Males, n (%)	14 (58%)	7 (58%)	7 (58%)	8 (73%)	8 (73%)	0.85
Diabetes mellitus, n (%)	0 (0%)	3 (25%)	3 (25%)	3 (27%)	3 (27%)	0.12
Cardiovascular disease, n (%)	0 (0%)	3 (25%)	na	3 (27%)	3 (27%)	0.043
eGFR^a^ (ml/min/1.73m^2^)	93.2 (80.3–105.4)	6.6 (5.7–8.2)	62.7 (44.9–74.3)	5.0 (3.9–9.0)	na	< 0.001
BMI (kg/m^2^)	23.7 (22.3–27.0)	23.8 (20.2–29.8)	24.6 (22.0–27.4)	25.5 (22.0–26.4)	26.0 (22.9–27.7)	0.61
**Biochemical characteristics**						
Albumin (g/l)	40 (38–42)	36 (34–37)	36 (34–39)	34 (32–40)	35 (34–40)	0.003
Creatinine (µmol/l)	75 (69–87)	680 (529–858)	117 (90–143)	742 (591–955)	761 (658–871)	< 0.001
Haemoglobin (g/l)	na	116 (105–126)	139 (129–145)	117 (101–126)	110 (106–126)	0.001
Total Cholesterol (mmol/l)	5.1 (4.5–5.8)	4.8 (4.4–6.3)	6.0 (4.8–7.7)	4.3 (4.0–5.2)	4.4 (4.1–5.3)	0.007
HDL-cholesterol (mmol/l)	na	1.3 (1.1–1.4)	1.2 (1.1–1.5)	1.5 (1.4–1.8)	1.5 (1.2–1.7)	0.11
Triglyceride (mmol/l)	1.0 (0.7–1.7)	1.8 (1.5–2.6)	2.6 (1.5–4.0)	1.5 (1.2–1.9)	1.6 (1.1–2.9)	0.022
IL-6 (pg/ml)	1.2 (0.7–3.4)	1.0 (0.8–1.3)	na	5.0 (3.7–9.5)	9.7 (4.4–14.9)	< 0.001
hsCRP (mg/l)	0.6 (0.4–1.7)	1.0 (0.2–3.5)	1.4 (0.8–9.5)	1.8 (1.0–4.5)	2.0 (1.0–2.7)	0.095
Leucocytes, (10^9^/l)	5.7 (4.7–7.0)	8.8 (6.5–10.5)	7.1 (6.0–7.4)	7.5 (4.1–8.0)	6.6 (4.7–7.6)	0.022
Thrombocytes, (10^9^/l)	257 (229–293)	240 (232–265)	211 (168–253)	238 (143–297)	263 (137–311)	0.70
Neutrophils, (10^9^/l)	2.8 (2.2–4.0)	6.7 (3.6–9.0)	4.4 (3.4–6.2)	4.1 (2.6–5.4)	3.2 (2.0–4.4)	0.002
Eosinophils, (10^9^/l)	0.3 (0.2–0.4)	0.1 (0.1–0.2)	0.1 (0.1–0.1)	0.3 (0.2–0.5)	0.2 (0.1–0.3)	0.002
Basophils, (10^9^/l)	0.04 (0.03–0.06)	0.1 (0.1–0.1)	0.1 (0.1–0.1)	0.1 (0.04–0.1)	0.1 (0.1–0.1)	< 0.001
Lymphocytes, (10^9^/l)	2.2 (1.7–2.7)	1.2 (1.0–1.4)	1.3 (0.9–2.0)	1.8 (0.9–2.1)	1.4 (0.9–2.0)	0.002
Monocytes, (10^9^/l)	0.3 (0.3–0.4)	0.6 (0.4–0.8)	0.7 (0.5–0.9)	0.5 (0.3–0.6)	0.4 (0.3–0.6)	< 0.001

Continuous variables are presented as median (25–75 percentile). Categorical variables are presented as number (n)/percentage (%). p-values are derived from statistical comparison (Chi square and Kruskal Wallis test) between all groups.

hsCRP high-sensitivity C-reactive protein, IL-6 interleukin-6, HDL high-density lipoprotein.

^a^eGFR, estimated GFR used the CKD-EPI equation.

### Clinical characteristics at 12 months follow-up after dialysis or transplantation

Follow-up data on patients are depicted in Table 1. As expected, in KTx patients, creatinine (680–117 µmol/l; p = 0.0005), leukocyte count (8.8–7.1 10^9^/l; p = 0.03), and neutrophil count (6.7–4.4 10^9^/l; p = 0.03) decreased, while body mass index (BMI) (23.8 to 24.6 kg/m^2^; p = 0.03) and haemoglobin levels (116–139 g/l; p = 0.002) increased after 12 months of follow-up. Apart from a statistically significant, but clinically non-important fall in eosinophil count (0.3–0.2 10^9^/l; p = 0.03), no significant changes in any of the measured clinical parameters were seen in dialysis patients after 12 months. The differences between controls and dialysis patients at 12 months remained, as did the differences between controls and KTx patients. In addition, hsCRP levels (1.4 vs. 0.6 mg/l, p = 0.04) were higher in KTx patients compared to controls. Comparing the two patient groups’ 12-month data, dialysis patients had, as expected, significantly higher creatinine (761 vs 117 µmol/l; p < 0.0001) and lower haemoglobin (110 vs 139 g/l; p = 0.001) as well as cholesterol (4.4 vs 6.0 mmol/l; p = 0.007) levels than KTx patients.

### Differentially methylated CpG sites before and after KFRT

**Figure 1:**
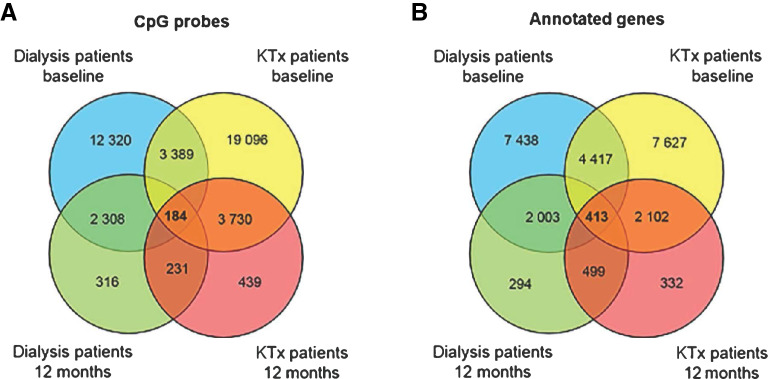
(**A**, **B**) Differentially methylated CpG sites in patients compared to controls. (**A**) Overlap and unique CpG sites including all significant sites; (**B**) overlap and unique CpG sites including sites associated with annotated genes. Statistical analysis performed with lumi. KTx kidney transplantation.

The Venn diagram in Fig. 1A illustrates the number of methylated CpG sites (compared to controls) that were different or common between patient groups at baseline and following 12 months of treatment (P_FDR_ < 0.01; increased or decreased degree of methylation). Overall, a larger number of unique CpG sites were found in KTx patients than in dialysis patients, especially at baseline (19096 CpG probes vs*.* 12320 CpG probes), but also after 12 months of treatment (439 CpG probes vs*.* 316 CpG probes). In the same manner, the number of annotated genes associated with significant CpG sites were compared between patient groups and over time in relation to controls (Fig. 1B). Approximately 7000 unique genes were identified at baseline, both in patients undergoing dialysis (7438 genes) and KTx (7627 genes). After treatment, the number of significant genes were reduced to 294 and 332 in dialysis and KTx patients, respectively. The intersection between patient groups at baseline and after KFRT in Fig. 1B demonstrated an overlap of 413 common genes. In contrast, none of the differentially methylated CpG sites reached statistical significance (i.e. P_FDR_ > 0.05) in the within group, pairwise, comparisons between patient samples at baseline and at 12 months of follow-up.

### Distribution pattern of differentially methylated CpG sites in relation to gene-related structures

**Figure 2:**
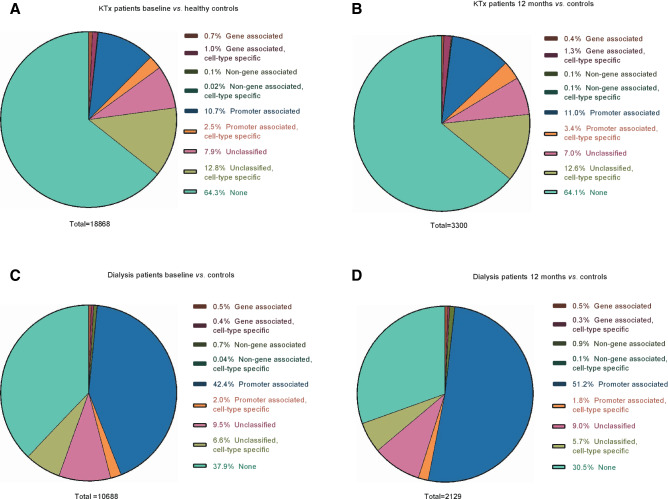
(**A**–**D**) Localisation of CpG sites with significantly different methylation compared to controls. (**A**) KTx patients at baseline versus controls; (**B**) KTx patients after 12 months versus controls; (**C**) dialysis patients at baseline versus controls; (**D**) dialysis patients after 12 months versus controls. KTx kidney transplantation.

**Figure 3:**
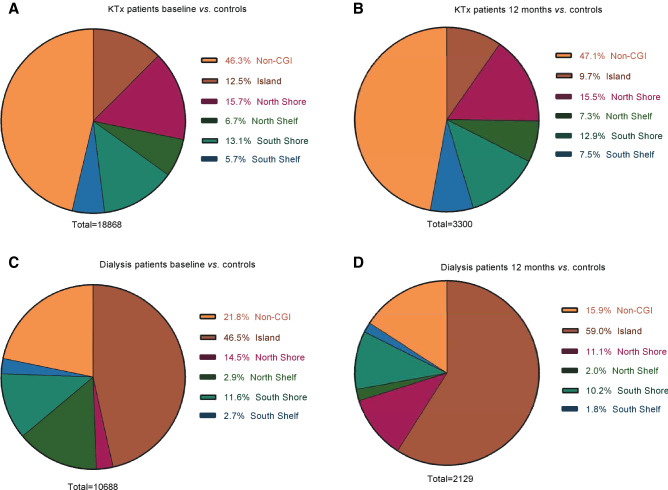
(**A**–**D**) Distribution of CpG sites within CpG islands with significantly different methylation compared to controls. (**A**) KTx patients at baseline versus controls; (**B**) KTx patients after 12 months versus controls; (**C**) dialysis patients at baseline versus controls; (**D**) dialysis patients after 12 months versus controls. CGI CpG island.

**Figure 4:**
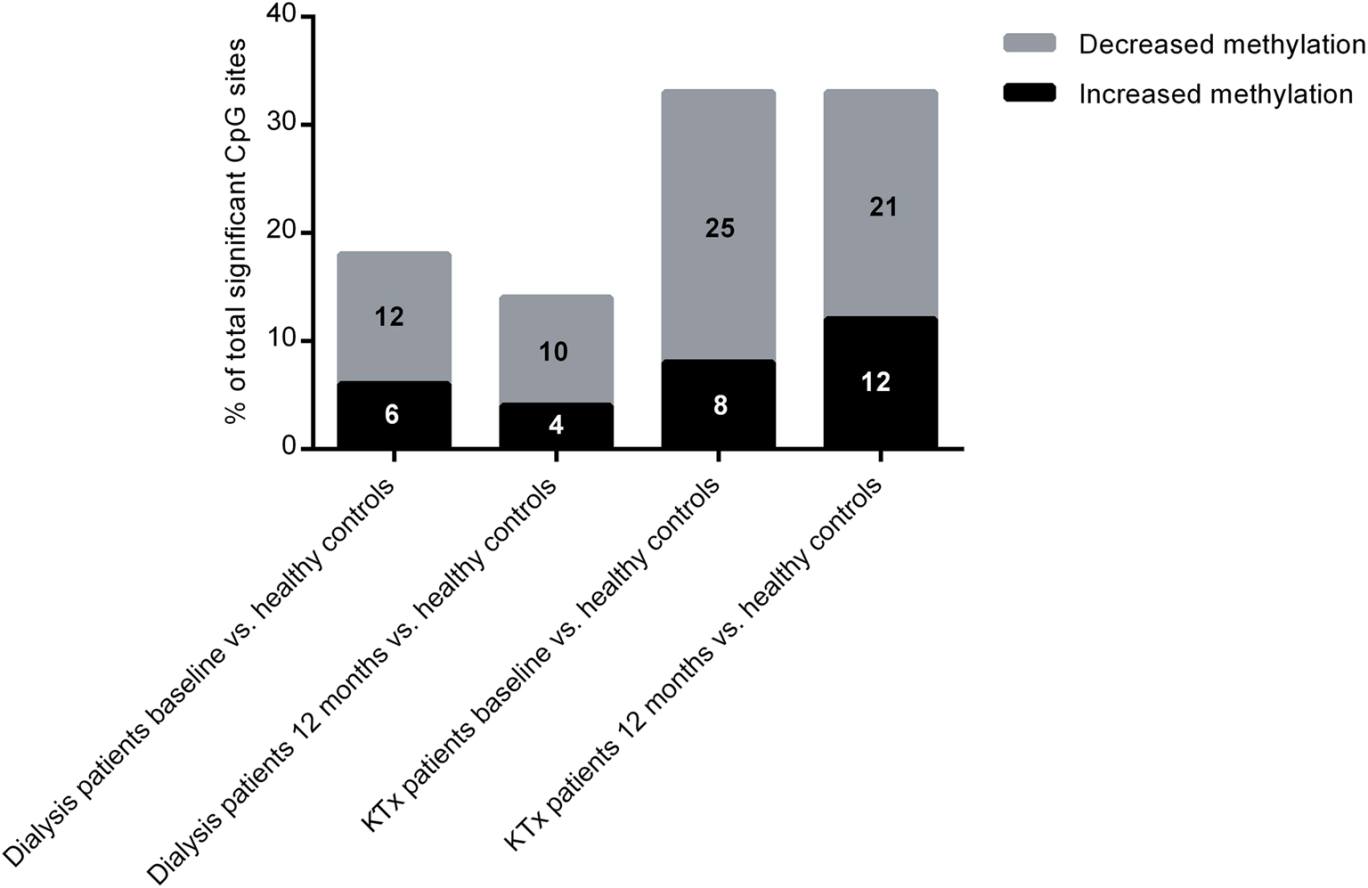
Percentage of significant CpG sites located in enhancer elements. Data is shown according to kidney replacement therapy group and time point. Numbers in bars indicate percentage. KTx kidney transplantation.

The distribution of differentially methylated CpG sites in gene-related structures is shown in Fig. 2A–D. A larger proportion of differentially methylated CpG sites in the KTx group was located in regions unrelated to genes (64% at baseline and 64% after 12 months; Fig. 2A,B) as opposed to the dialysis group, where the majority of sites were located in promoter associated regions (42% at baseline and 51% after 12 months; Fig. 2C,D). The distribution pattern of differentially methylated CpG sites with regard to CpG islands (CGIs) is shown in Fig. 3A–D. Again, the distribution profile was different between the two patient groups, as the majority of sites in KTx patients were not localized to any CGI (46% at baseline and 47% after 12 months; Fig. 3A,B), while most of the sites in dialysis patients were located within the island region (47% at baseline and 59% after 12 months; Fig. 3C,D). Furthermore, enhancers were more often differentially methylated in KTx patients compared to dialysis patients, both at baseline (33% vs 18%, respectively) and after 12 months (33% vs 14%, respectively) (Fig. 4).

### Genes associated with top 10 differentially methylated CpG sites

All the abbreviations and full official names of the genes discussed in this article can be found in Supplementary Table S1 online. All genes will be referred to by their official abbreviation only. Table 2 provides an overview of the 10 CpG sites from each patient group that showed the largest methylation differences (fold change) compared to healthy controls. In dialysis patients, CpG site cg12666727 in *HIVEP3* was significantly less methylated (fold change 0.05, P_FDR_ = 2.1 × 10^–4^) at baseline compared to healthy controls. In the same patient group, methylation of CpG site cg23813394 in the *COL6A3* gene was significantly increased (fold change 776.2, P_FDR_ = 7.8 × 10^–3^). After 12 months of dialysis treatment, CpG sites in *EID2* (site cg11976736; fold change 0.053, P_FDR_ = 5.5 × 10^–3^) and *ETS1* (site cg24065451; fold change 0.076, P_FDR_ = 8.7 × 10^–4^) were significantly less methylated in patients than in controls, whereas sites in *MRFAP1* (site cg24475210; fold change 39, P_FDR_ = 7.1 × 10^–4^) and *ZNF224* (site cg26649251; fold change 83.2, P_FDR_ = 1.4 × 10^–4^) displayed an increased degree of methylation.

**Table 2:** Top 10 CpG sites showing the largest fold change between patients and controls.

	Gene symbol	Probe no.	P_FDR_	Fold change
Controls vs dialysis baseline	*D2HGDH*	cg05385718	3.9 × 10^–4^	0.003
	*CDK6*	cg06688763	4.1 × 10^–4^	0.044
	*AGAP3*	cg25417842	4.1 × 10^–3^	0.045
	*PNKD*	cg22712983	1.4 × 10^–3^	0.049
	*HIVEP3*	cg12666727	2.1 × 10^–4^	0.05
	*HSDL1*	cg08965527	1.0 × 10–4	34.8
	*RNASEH2C*	cg25294185	1.2 × 10^–4^	36.3
	*ZNF562*	cg21610904	3.6 × 10^–3^	39.9
	*CSGALNACT2*	cg26674752	1.1 × 10^–4^	71.3
	*COL6A3*	cg23813394	7.8 × 10^–3^	776.2
Controls vs dialysis 12 months	*D2HGDH*	cg05385718	8.1 × 10^–3^	0.007
	*PNKD*	cg22712983	2.5 × 10^–3^	0.04
	*EID2*	cg11976736	5.5 × 10^–3^	0.053
	*AGAP3*	cg07429146	7.2 × 10^–3^	0.068
	*ETS1*	cg24065451	8.7 × 10^–4^	0.076
	*MRFAP1*	cg24475210	7.1 × 10^–4^	39
	*GBX1*	cg04553410	6.8 × 10^–4^	41.9
	*RNASEH2C*	cg25294185	3.3 × 10^–4^	44.7
	*ZNF224*	cg26649251	1.4 × 10^–4^	83.2
	–	cg12031275	7.4 × 10^–3^	288.5
Controls vs KTx baseline	*TTC15*	cg00257789	6.5 × 10^–3^	0.004
	*HLA-DRB5*	cg25372449	4.8 × 10^–3^	0.01
	*PNKD*	cg22712983	6.4 × 10^–6^	0.022
	*PRKAG2*	cg06436185	1.4 × 10^–6^	0.022
	*FXYD2*	cg01142676	1.8 × 10^–6^	0.027
	*BCL11B*	cg16452866	2.4 × 10^–6^	35.7
	*NOSIP*	cg21292909	3.3 × 10^–7^	38.4
	*IQSEC1*	cg26692003	4.4 × 10^–7^	41.9
	*DGKA*	cg07679948	3.6 × 10^–6^	45.2
	*RARG*	cg13937905	4.9 × 10^–6^	54.3
Controls vs KTx 12 months	*SSH3*	cg22632840	1.5 × 10^–4^	0.044
	–	cg13985485	3.5 × 10^–3^	0.054
	–	cg11799593	5.6 × 10^–3^	0.055
	*HOXD13*	cg04415176	3.5 × 10^–3^	0.057
	–	cg20697025	1.9 × 10^–3^	0.062
	*EHD1*	cg06154903	1 × 10^–4^	17.7
	*ACOT7*	cg11699125	1.6 × 10^–3^	18.1
	*LOC399815;FAM24B*	cg10241347	3.6 × 10^–4^	19
	*CDKL2*	cg05982271	6.5 × 10^–3^	19.3
	*RDH10*	cg17662034	2.2 × 10^–4^	24

The 10 probes with the largest positive and negative fold change (FC) from each patient group, using healthy controls as a reference, are shown.

FC range < 1 indicates less methylation in patients than in controls, while the opposite is true for FC range > 1.

FDR false discovery rate.

In KTx patients at baseline, CpG site cg06436185 in the *PRKAG2* gene was less methylated than in controls (fold change 0.022, P_FDR_ = 1.4 × 10^–6^). Also, sites in *BCL11B* (site cg16452866; fold change 35.7, P_FDR_ = 2.4 × 10^–6^) and *DGKA* (site cg07679948; fold change 45.2, P_FDR_ = 3.6 × 10^–6^) showed increased baseline methylation in patients vs. healthy controls.

The genes with the highest number of significant CpG sites in each patient group are presented in Table 3. In dialysis patients at baseline, the genes with the highest number of differentially methylated CpG sites included (but were not limited to) *SKI* (11 CpG sites), *PRDM16* (10 CpG sites), *HDAC4* (8 CpG sites), *ZMIZ1* (7 CpG sites), *GFI1* (7 CpG sites), and *HIVEP3* (7 CpG sites). After 12 months of dialysis treatment, genes with the highest number of differentially methylated CpG sites compared to controls included *MAD1L1* (5 CpG sites), *MRFAP1* (3 CpG sites), *HDAC4* (3 CpG sites), and *SKI* (3 CpG sites). *SKI, PRDM16,* and *HDAC4* all had several CpG sites that remained differentially methylated over time compared to controls.

**Table 3:** Genes with the highest number of statistically significant probes (FDR < 0.01).

	Gene symbol	CpG sites	P_FDR_ range	FC range
Controls vs dialysis baseline	*SKI*	11	1.5 × 10^–4^ to 9.7 × 10^–3^	0.21–4.07
	*PRDM16*	10	6.6 × 10^–4^ to 8.7 × 10^–3^	0.21–2.51
	*TRIM26*	8	2.5 × 10^–4^ to 8.8 × 10^–3^	0.19–5.75
	*HDAC4*	8	2.8 × 10^–4^ to 9.8 × 10^–3^	0.19–12.88
	*ZMIZ1*	7	2.6 × 10^–4^ to 9.2 × 10^–3^	0.2–0.58
	*LOC146880*	7	3.2 × 10^–4^ to 9.8 × 10^–3^	0.17–0.5
	*GFI1*	7	1.1 × 10^–4^ to 8 × 10^–3^	0.08–0.24
	*HIVEP3*	7	1.7 × 10^–4^ to 8.1 × 10^–3^	0.05–2.82
	*BAHCC1*	7	7 × 10^–4^ to 8.7 × 10^–3^	0.32–0.46
Controls vs dialysis 12 months	*MAD1L1*	5	5.4 × 10^–4^ to 7.5 × 10^–3^	0.26–0.42
	*MYL9*	4	2.9 × 10^–3^ to 6.1 × 10^–3^	0.25–0.37
	*LOC284837*	4	4.6 × 10^–4^ to 3.5 × 10^–3^	0.33–0.45
	*SKI*	3	1 × 10^–3^ to 8.9 × 10^–3^	0.28–3.8
	*PTPRCAP*	3	6.7 × 10^–3^ to 7.2 × 10^–3^	1.86–2.82
	*PRDM16*	3	2.6 × 10^–3^ to 5.9 × 10^–3^	0.32–0.47
	*MRFAP1*	3	7.1 × 10^–4^ to 6.6 × 10^–3^	2.69–38.9
	*RPS11*	3	3.3 × 10^–3^ to 6 × 10^–3^	3.63–17.78
	*FANCF*	3	2.9 × 10^–3^ to 8.4 × 10^–3^	2.51–3.39
	*HDAC4*	3	1.3 × 10^–4^ to 4.4 × 10^–3^	2.57–5.5
	*C17orf64*	3	8.8 × 10^–4^ to 9.7 × 10^–3^	0.19–0.46
Controls vs KTx baseline	*MAD1L1*	34	4.6 × 10^–7^ to 9.2 × 10^–3^	0.11–22.91
	*HDAC4*	29	6.4 × 10^–6^ to 8.9 × 10^–3^	0.07–14.45
	*RPTOR*	28	1.2 × 10^–6^ to 5.1 × 10^–3^	0.07–9.77
	*TBCD*	26	1.5 × 10^–6^ to 7.4 × 10^–3^	0.05–21.38
	*NCOR2*	26	1.5 × 10^–6^ to 8.4 × 10^–3^	0.07–2.69
	*RASA3*	22	4.8 × 10^–7^ to 9.1 × 10^–3^	0.05–9.12
	*KCNQ1*	20	4.1 × 10^–6^ to 9.7 × 10^–3^	0.15–18.62
	*ANKRD11*	19	4.4 × 10^–7^ to 8.9 × 10^–3^	0.09–7.41
	*BCL11B*	18	4.7 × 10^–7^ to 2.7 × 10^–3^	0.3–35.48
Controls vs KTx 12 months	*RASA3*	11	2.9 × 10^–4^ to 9.5 × 10^–3^	0.09–5.62
	*MAD1L1*	11	6.9 × 10^–4^ to 9.8 × 10^–3^	0.2–10.23
	*TBCD*	9	4.9 × 10^–4^ to 9.2 × 10^–3^	0.13–4.79
	*RPTOR*	9	7.3 × 10^–4^ to 9.2 × 10^–3^	0.15–3.89
	*DDR1*	8	3.3 × 10^–4^ to 7.1 × 10^–3^	1.78–5.37
	*BANP*	8	2.4 × 10^–4^ to 6.2 × 10^–3^	0.19–5.89
	*ARID3A*	8	9 × 10^–4^ to 8.8 × 10^–3^	0.06–0.32
	*FOXK1*	7	4.8 × 10^–3^ to 9.3 × 10^–3^	0.12–2.95
	*ITPKB*	7	6.9 × 10^–4^ to 9.6 × 10^–3^	0.32–6.31
	*HDAC4*	7	6.5 × 10^–4^ to 4.4 × 10^–3^	0.19–10.23

FC range < 1 indicates less methylation in patients than in controls, while the opposite is true for FC range > 1.

FDR false discovery rate, FC fold change, using healthy controls as a reference.

Similarly to that observed in the dialysis group, *MAD1L1* and *HDAC4* had the highest number of differentially methylated CpG sites when comparing KTx patients at baseline to healthy controls (34 CpG sites and 29 CpG sites, respectively). Other genes that showed a high number of differentially methylated CpG sites in the KTx group were *RPTOR* (28 CpG sites), *TBCD* (26 sites), *NCOR2* (26 CpG sites), *RASA3* (22 sites), *KCNQ1* (20 CpG sites), *ANKRD11* (19 CpG sites), and *BCL11B* (18 CpG sites). Twelve months after KTx, CpG sites in *RASA3, MAD1L1, RPTOR, TBCD* and *HDAC4* all remained significantly different compared to healthy controls (11 CpG sites, 11 CpG sites, 9 CpG sites, 9 CpG sites and 7 CpG sites, respectively). Additional genes included *DDR1* (8 CpG sites), *BANP* (8 CpG sites) and *ARID3A* (8 CpG sites).

Overall, the number of significant CpG sites were reduced in each group after 12 months of KFRT. *HDAC4* was the only gene that showed a high number of significant CpG sites in both dialysis and KTx patients, at baseline (8 and 29 CpG sites, respectively) as well as after 12 months (3 and 7 CpG sites, respectively). After 12 months, significant CpG sites in *MAD1L1* were observed in both patient populations (5 CpG sites in dialysis patients and 11 sites in KTx patients)*.*

### Pathway enrichment analysis

Canonical pathways displaying the largest statistical significance after separate core analyses of both patient groups at both time points are shown in Table 4. All differentially methylated CpG sites compared to healthy controls, both at baseline and after 12 months of KFRT, maintained the same profile with regards to being hypo- or hypermethylated. Pathway enrichment analysis results from core analysis of the 413 genes that contained at least one differentially methylated CpG site in both patient groups at both time points are also shown.

**Table 4:** Top ranked canonical pathways from IPA after performing basic core analysis.

	Canonical pathway title	p-value
Controls vs dialysis patients baseline	Protein ubiquitination pathway	3.8 × 10^–10^
	Cell cycle: G1/S checkpoint regulation	2 × 10^–6^
	Role of BRCA1 in DNA damage response	1.1 × 10^–5^
	Cell cycle control of chromosomal replication	1.3 × 10^–5^
	Cell cycle: G2/M DNA damage checkpoint regulation	4.1 × 10^–5^
	RAR activation	8.1 × 10^–5^
Controls vs KTx patients baseline	Molecular mechanisms of cancer	1.3 × 10^–13^
	Axonal guidance signalling	3.1 × 10^–10^
	Integrin signalling	6.9 × 10^–9^
	Fcγ receptor-mediated phagocytosis in macrophages and monocytes	1.5 × 10^–8^
	Protein kinase A signalling	1.7 × 10^–8^
	G-protein coupled receptor signalling	3 × 10^–8^
Controls vs dialysis patients 12 months	Cyclins and cell cycle regulation	1.6 × 10^–7^
	Cell cycle: G1/S checkpoint regulation	3.5 × 10^–7^
	Small cell lung cancer signalling	2.9 × 10^–6^
	Prostate cancer signalling	2 × 10^–5^
	Chronic myeloid leukemia signalling	5.5 × 10^–5^
	Molecular mechanisms of cancer	1.3 × 10^–4^
Controls vs KTx patients 12 months	Integrin signalling	9.3 × 10^–9^
	Molecular mechanisms of cancer	1 × 10^–8^
	Paxillin signalling	1.4 × 10^–7^
	Tec kinase signalling	6.5 × 10^–7^
	T cell receptor signalling	9.4 × 10^–7^
	Role of NFAT in regulation of the immune response	1.1 × 10^–6^
Controls vs all^a^	IGF-1 signalling	3.3 × 10^–5^
	Rac signalling	5.5 × 10^–5^
	STAT3 pathway	1.3 × 10^–4^
	Breast cancer regulation by Stathmin1	1.7 × 10^–4^
	PTEN signalling	1.7 × 10^–4^

^a^Gene affiliations from all CpG sites displaying statistically significant differentially methylation in patients versus controls at both time points were entered into the analysis (see main text for more information).

## Discussion

We studied the global CpG methylation pattern in two groups of KF patients starting KFRT—patients initiating dialysis treatment and patients undergoing KTx—and compared it to the methylation profile in an age- and sex-matched population-based control group. We uncovered disease-specific DNA methylation differences prior treatment and identified genomic regions where the CpG methylation profile was modified 12 months after start of KFRT. KFRT changed the methylation profiles of regions associated with cellular ageing and metabolic processes so that they resembled those observed in healthy controls. In addition, our study design enabled us to identify genes that may be specifically associated with CKD, as their methylation status compared to healthy controls remained constant over time in both CKD groups and despite treatment modality. To the best of our knowledge, this is the first study to longitudinally investigate the impact of KFRT on CpG methylation.

Analyses exposed more than 12000 and 19000 methylated CpG sites unique to dialysis and KTx patients at baseline, respectively. Several genes with functional profiles relevant to CKD and its comorbidities were identified. Numerous genes have been shown to be closely involved in apoptotic and proliferative cellular processes, which is interesting given the established links between apoptosis, vascular calcification and CVD^10–12,29,30^. *HIVEP3*^31^, *EID2*^32^, *ETS1*^33^, *MRFAP1*^34^, *ZNF224*^35^, *BCL11B *^36,37^, *SKI*^38,39^, *ZMIZ1*^40^, *GFI1*^41^, *HDAC4*^42^, *MAD1L1*^43^, *NCOR2*^44,45^, *ANKRD11*^46^, *BANP*^47^, and *ARID3A*^48,49^ all have functions related to apoptosis, cell survival or cell cycle progression. This highlights the importance of cell cycle activity, apoptosis, and cellular senescence in CKD. Allostatic overload that increases cellular age and stress, such as oxidative stress, inflammation, and uremic toxins, is a hallmark of the uremic milieu^50,51^. Thus, it could be hypothesised that the disease itself accelerates cellular ageing, and that this is reflected in the altered methylation status of genes involved in biological ageing processes^7^. Several of the annotated genes have previously been associated with CKD, CVD and/or diabetes. Among these are *COL6A3*^52,53^, *PRKAG2 *^54–56^, *PRDM16 *^57–59^, *KCNQ1*^60,61^, *RPTOR*^62^, *DDR1*^63,64^, and *BCL11B*^65^. In addition, a number of genes linked to inflammation were identified, i.e. *DGKA*^66^, *COL6A3*^52,53^, *HDAC4*^67^, *NCOR2*^68^, and *DDR1*^63^. It is well-known that persistent inflammation is associated with CVD, DM^69^, cellular ageing^7,70^, and CKD^71^. The genes *HDAC4, COL6A3*, *DDR1*, *PRKAG2*, *BCL11B*, and *NCOR2* are involved in two or more of the clinical phenotypes mentioned above and warrant particular attention*.* We also identified genes that have not previously been associated with CKD, such as *HIVEP3*, which has the ability to attenuate vascular smooth muscle cell (VSMC) transition and the capacity to reduce tissue calcification^72^. Taken together, differences in methylation profiles between KFRT patients and controls were often present in genes and regions known to be relevant to the uremic phenotype. Although the risk factor profile differed comparing incident dialysis patients and patients selected for KTx, these differences were not observed when comparing the two different KFRT groups to each other.

Comparisons with candidate genes reported in previous epigenome-wide analyses, addressing CKD and kidney function, reveal partly overlap with the top-ranked genes found in this study. In a large epigenome-wide study on whole-blood DNA, differentially methylated *PRKAG2, ANKRD11* and *BANP* loci were observed in patients with CKD^25^. Interestingly, *PRKAG2* also associated with renal function and CKD in a meta-analysis of genome-wide data^73,74^ and was differentially methylated in kidney tissue in a study comparing methylation profiles between individuals with and without diabetic kidney disease (DKD)^75^. In another study on kidney tissue (tubules) from patients with DKD, CpG methylation in *ANKRD11* as well as *ARID3A, SKI, BCL11B, MRFAP, ETS11* and *KCNQ1* loci were correlated with gene expression levels, supporting a functional role of DNA methylation^22^. Moreover, epigenome-wide data on whole-blood DNA suggests that methylated CpGs at *ANKRD11*, mapping to active enhancers in kidney cortex, may be important in relation to kidney function^21^. In diabetic Pima Indians, *MAD1L1* was one of the top 20 probes at which methylation levels associated with development of KF^23^. A recent study identified *COL6A3* as being differentially methylated*,* along with other genes involved in the production and deposition of the extracellular matrix, in uremic arterial tissue^76^.

Following 12 months of KFRT, the number of differentially methylated CpG sites was markedly reduced in both patient groups (80% reduction in dialysis patients and 82% reduction in KTx patients) compared to controls. Thus, although we could not identify any statistically significant differences in the degree of methylation of CpG probes/annotated genes when performing paired analyses over time within the patient groups, KFRT appears to normalise methylation differences seen between KF patients and controls. This implies that not only does the toxic uremic milieu itself impose large-scale alterations in the epigenetic profile of the DNA molecule, but also that these changes can be at least partially reversed by KFRT.

The normalisation process observed after KFRT occurred rather uniformly across the different types of CpG sites in the genome, as the distribution profile of differentially methylated CpG sites at regulatory elements, CGI components, or enhancers remained similar over time within the two KFRT groups (Figs. 2A–D, 3A–D and 4). Interestingly, the inter-group distribution pattern of the CpG methylation sites was markedly different among patients already before initiating treatment. While the underlying reasons are unknown, it is worth considering that patients undergoing KTx constitute a selected group with better general health status and improved long-term survival than patients that remain on dialysis^3^. Although this is reflected by significantly higher levels of IL-6 in dialysis patients, both before and after 12 months of KFRT, indicating an activation of the SASP^77^, other factors not accounted for such as steroid medication could play a role. The SASP response, including secretion of large amounts of bioactive molecules, such as IL-6 from senescent cells, drives the development of senescence-related inflammation, metabolic dysregulation, stem cell dysfunction and other ageing phenotypes^78^. Plasma levels of IL-6, and other pro-inflammatory molecules, associate with whole-blood DNA methylation signatures in complex diseases and ageing^79–82^. Moreover, inflammation and ageing are mechanistically linked with changes in the DNA methylome^28,83^.

Importantly, we identified a set of 413 differentially methylated genes at baseline in both patient groups that remained significant over 12 months of KFRT, indicating that these genes may be characteristic of CKD in general and are unaffected by treatment. Contrary to those genes whose methylation status was changed after 12 months of KFRT, this group of genes may include viable candidates for identifying underlying factors involved in progression to KF. Notably, the pathway analysis of this core group of genes revealed several significant canonical pathways of biological interest in this context, such as IGF-1 signalling, the STAT3 pathway, Rac signalling, and PTEN signalling. IGF-1 signalling is a regulator of biological ageing and modulates resistance to oxidative stress^84^. In fact, STAT3 is induced by IGF-1 signalling^85^. STAT3 has also been shown to associate with Rac1 GTPase^86^, a signalling pathway associated with podocyte damage and a number of cardiac diseases^87^. There is also an obvious link to DM and insulin resistance, as IGF-1 receptor signalling—and thereby also STAT3 and Rac signalling—not only occurs through IGF-1, but also through insulin^88^. Meanwhile, loss of the tumour suppressor gene *PTEN* induces cellular senescence^89^. In a mouse model of CKD, Xu et al*.*^90^ found that downregulation of PTEN occurs as a protective mechanism against muscle wasting and moreover, *PTEN* promoter methylation has been associated with the presence of metabolic syndrome in humans^91^. In addition, the most significantly associated clinical feature with this set of genes was CVD (p-value 5.7 × 10^–3^ to 1.9 × 10^–7^), the most prominent mortality factor in CKD^5,6^. Taken together, our findings suggest that biological ageing processes, insulin/IGF signalling, and oxidative stress may be central components of KF and its main comorbidity CVD.

This study provides an overall, genome-wide view of methylation status in KF, which may enable future identification of pathways that are responsible for disease or surrogate biomarkers. Epigenome-wide information in combination with genome-wide expression analysis opens up opportunities for pharmacological editing of DNA modifications as a potential treatment strategy^92–94^. In addition, information on disease captured via methylation markers in blood or urine could be used as a non-invasive screening tool to follow disease progression and severity in CKD. Recently, it has been shown that urinary methylation levels were able to predict a 30% decline in the estimated GFR, or development of KF over a period of three years, when combined with macroalbuminuria or an increased level of Alpha 1 Microglobulin^95^. Finally, data showing that cholesterol lowering treatment restores blood DNA methylation in patients with CKD stages 3–4 suggest that methylation could also be a functional and relevant measurement of treatment response^96^.

Some caveats of the study need consideration. One shortcoming is the small number of patients studied, limiting the possibility to adjust for multiple confounders, including underlying diagnosis, mode of dialysis and cell type composition, as well as hindering robust conclusions being made from the results. Lack of validation cohort further limits our results. Thus, data presented in this paper should be used to design larger confirmatory studies. In this context, it is also worth noting that this study does not allow disentangling the putative effects on global methylation caused by the disease itself from effects due to medication present before obtaining the baseline sample—e.g. immunosuppressive treatment given to patients undergoing KTx (see “Methods” section). Indeed, the effect of steroid treatment on DNA methylation is largely unknown and deserves further study^97^.

To conclude, this is the first longitudinal study of effects of KFRT on genome-wide CpG methylation in KF. The differences in genome-wide methylation profiles between KF patients and controls were reduced after KFRT, pointing towards a normalising of the epigenome. The strikingly different distribution of significant CpG sites between the two patient populations suggests that there is an underlying epigenetic difference between patients remaining on dialysis and those selected for KTx. This observation is of particular interest as patients on the KTx waiting list have a better outcome than those who are to undergo dialysis. As IL-6 was the only identified factor that was consistently different between the two groups, difference in allostatic load and persistent inflammation may play a role. Genes with CpG sites that were statistically significant in both patient groups at both time points indicate a prominent involvement of processes related to cell growth, cell proliferation, and/or the cell cycle. Numerous significant CpG sites were also located in genes that have previously been associated with CKD and/or its comorbidities, such as CVD and DM. In summary, our results contribute to current knowledge by illuminating an epigenetic signature with genes and pathways that differs between KF patients and the general population and hold potential to pinpoint DNA methylation changes underlying the clinical effects observed with KFRT.

## Methods

### Ethics declarations

The study was approved by the regional Swedish Ethical Review Authority in Stockholm (approval no 2016/1470-31/4, 2008/1748-31/2 and 40/02) and adhered to the Principles of the Declaration of Istanbul as outlined in the “Declaration of Istanbul on Organ Trafficking and Transplant Tourism”, as well as to the Helsinki declaration. All subjects included in the present study provided written informed consent.

### Dialysis patients

Twelve patients initiating dialysis at the Department of Renal Medicine, Karolinska University Hospital, Stockholm, Sweden, with median age 48 years and 8 (73%) males, were included. One patient was subsequently excluded from downstream analyses due to insufficient DNA methylation signal quality, resulting in a total of 11 dialysis patients in the study. Blood samples were drawn at a time point close to start of dialysis, and again after 12 months of dialysis. Eight patients received peritoneal dialysis, and four were treated by haemodialysis. Underlying causes of CKD were suspected diabetic nephropathy (n = 3; 27%), chronic glomerulonephritis (n = 2; 18%), adult polycystic kidney disease (n = 1; 9%) and CKD of unknown or other causes (n = 5; 45%). Three patients (27%) had DM and three (27%) had CVD (clinical signs of cerebrovascular, cardiovascular, and/or peripheral vascular disease) at baseline. Other comorbidities included infectious disease (two patients), hemochromatosis (one patient), ulcerous colitis (one patient) and polymyalgia (one patient). Common medications in the dialysis-treated group included ACE-inhibitors/angiotensin receptor blockers (91%), diuretics (91%), vitamin D (91%) and phosphate binders (82%).

### Kidney transplant patients

Twelve patients undergoing KTx at the Department of Transplantation Surgery, Karolinska University Hospital, Stockholm, Sweden were included in this study. Median age in this group was 48 years and 7 patients (58%) were males. Ten patients (83%) received transplants from living donors, while two (17%) received transplants from deceased donors. Blood samples were drawn immediately before surgery, and again 12 months later. Among KTx patients, three (25%) had been treated with haemodialysis (median vintage 0.3 [0.2–4] months) and four (33%) had been treated with peritoneal dialysis (median vintage 1 [0.4–2.6] months) prior to KTx. Underlying causes of chronic kidney disease included suspected diabetic nephropathy (n = 2; 17%), chronic glomerulonephritis (n = 1; 8%), adult polycystic kidney disease (n = 3; 25%), and CKD of other or unknown cause (n = 6; 50%). At baseline, three patients (25%) had DM, and three patients (25%) had CVD. One patient had undergone kidney transplantation 19 years earlier, two had been treated for, and cured from, cancer (squamous cell carcinoma and thyroid cancer, respectively), and one patient suffered from rheumatoid arthritis. Two days prior to surgery, all patients displaying blood group compatibility (n = 9; 75%) received tacrolimus, prednisolone and mycophenolate mofetil (MMF). None of the patients received treatment with mTOR inhibitors. Three patients (25%) displayed blood group incompatibility. These patients received rituximab four weeks prior to KTx, and MMF and prednisolone medication was started 10 days before KTx according to the protocol for blood group incompatibility at the transplantation unit. Methylprednisolone (Solu-Medrol^®^) was administered on the day of KTx. Tacrolimus, prednisolone and MMF were given similarly to the routine in blood group compatible patients from one day post-surgery. In addition to these medications, common drugs in the KTx group included active vitamin D (92%), non-calcium based phosphate binders (83%), loop-diuretics (83%) and erythropoietin (75%).

### Control subjects

A randomly selected, population-based cohort was recruited by Statistics Sweden (SCB). The only exclusion criterion was unwillingness to participate. Of these, 24 individuals were selected to match for age and sex with patients in the dialysis and transplantation group. The median age of the controls was 52 years, and 14 individuals (58%) were male. Glomerular filtration rate (GFR), as evaluated with iohexol clearance, was 94 (84–102) ml/min/1.73 m^2^. None of the controls had known DM or CVD. Controls were investigated according to a protocol similar to the one utilised for the patient groups.

### Biochemical analyses

Venous blood samples for laboratory tests were collected after an overnight fast as described above and stored at − 80 °C pending analyses. Biochemical measurements (albumin, creatinine, haemoglobin, high-sensitive CRP (hsCRP), cholesterol, triglycerides) and blood cell counts (leukocytes, neutrophils, eosinophils, lymphocytes, monocytes) were conducted at the accredited Clinical Chemical Laboratory Lab at the Karolinska University Hospital, Stockholm, Sweden. Interleukin 6 (IL-6) was analysed by an immunometric assay on an Immulite 1000 Analyzer (Siemens Healthcare Diagnostics, Los Angeles, CA, USA) according to standard protocol.

### DNA isolation

DNA was isolated from whole blood using QIAamp DNA blood maxi kit (Qiagen, Hilden, Germany), and DNA concentration and integrity were assessed by NanoDrop ND-1000 (NanoDrop, Wilmington, DE, USA). All procedures were performed in accordance with the manufacturers’ protocols.

### Illumina infinium HumanMethylation450 BeadChip analysis

Bisulfite conversion of DNA samples (500 ng) was performed using EZ-96 DNA Methylation kit (Zymo Research, Irvine, CA, USA), followed by quantitative genome-wide DNA methylation analysis using the Illumina Infinium HumanMethylation450 BeadChip (Illumina, San Diego, CA, USA). All laboratory procedures were performed in accordance with instructions from the manufacturers. Methylation data was visualized using GenomeStudio software version 2011.1 (Illumina Inc.). Level of methylation was defined as the signal intensity of methylated alleles divided by the sum of the intensity signals of methylated + unmethylated alleles. The resulting β value ranged from 0 (representing no methylation) to 100 (representing 100% methylation).

### Statistical analyses

Patient characteristics and biochemistry are presented as median and interquartile range. To test statistical differences between groups, Χ^2^ test, Fisher’s exact test (nominal variables) or non-parametric Kruskal Wallis or Wilcoxon rank sum test (continuous variables) were applied, using software JMP^®^ 14.0.0 (SAS Institute Inc., North Carolina, USA). Paired analysis (Wilcoxon signed rank test) was used when comparing baseline and 12-month data from the same individual. Data from Illumina Infinium HumanMethylation450 BeadChip was normalised using quantile colour adjustment and analysed with the lumi package in Bioconductor^98^. Following lumi analysis, the data was further processed using the limma package^99^. In limma, the following comparisons were made: dialysis patients at baseline vs healthy controls; dialysis patients after 12 months vs healthy controls; KTx patients at baseline vs healthy controls; KTx patients after 12 months vs healthy controls; dialysis patients at baseline vs dialysis patients after 12 months; and KTx patients at baseline vs KTx patients after 12 months. False discovery rate (FDR) was calculated using Benjamini-Hochberg, and statistical significance was set at P_FDR_ < 0.01. All significant probes overlapping DNA sequences with known single nucleotide polymorphisms (SNPs) were removed from further analysis. Information on regulatory elements such as enhancers and CGIs were incorporated into the analysis.

### Pathway enrichment analysis

After performing initial statistical analysis (lumi and limma), significant Illumina Infinium HumanMethylation450 BeadChip probes with gene annotations were entered into QIAGEN’s Ingenuity^®^ Pathway Analysis (IPA^®^, QIAGEN Redwood City, www.qiagen.com/ingenuity), using control subjects as a reference group. Core analysis was used to identify pathways and networks in which a larger number of genes than could be expected by chance could be found among our candidate genes. For probes with more than one gene annotation, all annotations were entered separately, with the same P_FDR_ and fold change values. In addition, core analysis of a subset of 413 genes that contained at least one differentially methylated CpG site in both patient groups at both time points was performed.

## Supplementary Information

Supplementary Table S1.

## Data Availability

The dataset supporting the conclusions of this article is available in the ArrayExpress repository, accession number E-MTAB-4931 (http://www.ebi.ac.uk/arrayexpress/experiments/E-MTAB-4931). Additional data generated or analysed during this study are included in this published article (and its Supplementary Information files).
