# Supplementary material for: Longitudinal genome-wide DNA methylation changes in response to kidney failure replacement therapy
Source: Sci Rep. 2022 Jan 10;12:470. doi: 10.1038/s41598-021-04321-5 (PMC8748627; doi:10.1038/s41598-021-04321-5)
Supplement: Supplementary file 1 — Supplementary Table S1. [file 41598_2021_4321_MOESM1_ESM.docx]

**Supplementary Information**

**LONGITUDINAL GENOME-WIDE DNA METHYLATION CHANGES IN RESPONSE TO KIDNEY FAILURE REPLACEMENT THERAPY**

**Running Title: Longitudinal DNA methylation in kidney failure replacement therapy patients**

Anna Witasp^1^ (anna.witasp@ki.se), Karin Luttropp^2,3^ (karin.luttropp@evidera.com), Abdul Rashid Qureshi^1^ (tony.qureshi@ki.se), Peter Barany^1^ (peter.barany@ki.se), Olof Heimbürger^1^ (olof.heimburger@ki.se), Lars Wennberg^4^ (lars.wennberg@sll.se), Tomas J. Ekström^2,3^ (tomas.ekstrom@ki.se), Paul G Shiels^5^ (paul.shiels@glasgow.ac.uk), Peter Stenvinkel^1^ (peter.stenvinkel@ki.se), Louise Nordfors^1^***** (louise.nordfors@telia.com)

**Supplementary Table S1. Gene name abbreviations and full names in alphabetical order**

| **Abbreviation** | **Full name** |
| --- | --- |
| *ANKRD11* | Ankyrin repeat domain-containing protein 11 |
| *ARID3A* | AT-rich interaction domain-containing protein 3A |
| *BANP* | BTG3-associated nuclear protein |
| *BCL11B* | BAF chromatin remodeling complex subunit BCL11B |
| *COL6A3* | Collagen, type IV, alpha 3 |
| *D2HGDH* | D-2-hydroxyglutarate dehydrogenase |
| *DDR1* | Discoidin domain receptor family, member1 |
| *DGKA* | Diacylglycerol kinase, alpha |
| *EID2* | EP300-interacting inhibitor of differentiation 2 |
| *ETS1* | ETS protooncogene, transcription factor |
| *FXYD2* | FXYD domain-containing ion transport regulator 2 |
| *GFI1* | Growth factor-independent 1 |
| *HDAC4* | Histone deacetylase 4 |
| *HIVEP3* | Human immunodeficiency virus type I enhancer binding protein 3 |
| *HSDL1* | Hydroxysteroid dehydrogenase-like protein 1 |
| *IGF1* | Insulin-like growth factor 1 |
| *KCNQ1* | Potassium channel, voltage-gated, KQT-like subfamily, member 1 |
| *MAD1L1* | Mitotic arrest deficient 1like 1 |
| *MRFAP1* | MORF4 family-associated protein 1 |
| *NCOR2* | Nuclear receptor corepressor 2 |
| *NOSIP* | Nitric oxide synthase-interacting protein |
| *PNKD* | PNKD metallo-beta-lactamase domain-containing protein |
| *PRDM16* | PR domain-containing 16 |
| *PRKAG2* | Protein kinase, AMP-activated, non-catalytic, gamma-2 |
| *PTEN* | Phosphatase and tensin homolog |
| *RARG* | Retinoic acid receptor, gamma |
| *RASA3* | RAS p21 protein activator 3 |
| *RNASEH2C* | Ribonuclease H2, subunit C |
| *RPTOR* | Regulatory associated protein of MTOR |
| *SKI* | SKI protooncogene |
| *STAT3* | Signal transducer and activator of transcription 3 |
| *ZMIZ1* | Zinc finger MIZ-domain containing 1 |
| *ZNF224* | Zinc finger protein 224 |
